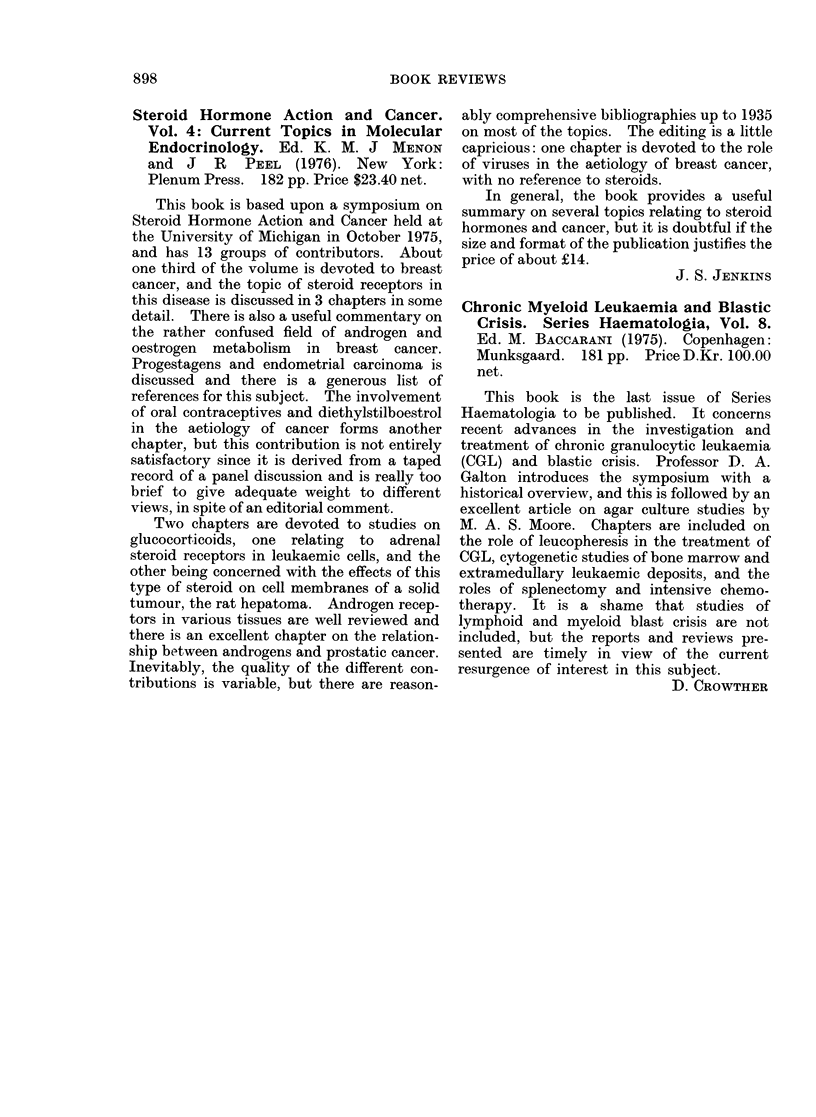# Steroid Hormone Action and Cancer. Vol. 4: Current Topics in Molecular Endocrinology

**Published:** 1977-06

**Authors:** J. S. Jenkins


**Steroid Hormone Action and Cancer. Vol. 4: Current Topics in Molecular Endocrinology.** Ed. K. M. J Menon and J R Peel (1976). New York: Plenum Press. 182 pp. Price $23.40 net.

This book is based upon a symposium on Steroid Hormone Action and Cancer held at the University of Michigan in October 1975, and has 13 groups of contributors. About one third of the volume is devoted to breast cancer, and the topic of steroid receptors in this disease is discussed in 3 chapters in some detail. There is also a useful commentary on the rather confused field of androgen and oestrogen metabolism in breast cancer. Progestagens and endometrial carcinoma is discussed and there is a generous list of references for this subject. The involvement of oral contraceptives and diethylstilboestrol in the aetiology of cancer forms another chapter, but this contribution is not entirely satisfactory since it is derived from a taped record of a panel discussion and is really too brief to give adequate weight to different views, in spite of an editorial comment.

Two chapters are devoted to studies on glucocorticoids, one relating to adrenal steroid receptors in leukaemic cells, and the other being concerned with the effects of this type of steroid on cell membranes of a solid tumour, the rat hepatoma. Androgen receptors in various tissues are well reviewed and there is an excellent chapter on the relationship between androgens and prostatic cancer. Inevitably, the quality of the different contributions is variable, but there are reasonably comprehensive bibliographies up to 1935 on most of the topics. The editing is a little capricious: one chapter is devoted to the role of viruses in the aetiology of breast cancer, with no reference to steroids.

In general, the book provides a useful summary on several topics relating to steroid hormones and cancer, but it is doubtful if the size and format of the publication justifies the price of about £14.

J. S. Jenkins